# The growing use of herbal medicines: issues relating to adverse reactions and challenges in monitoring safety

**DOI:** 10.3389/fphar.2013.00177

**Published:** 2014-01-10

**Authors:** Martins Ekor

**Affiliations:** Department of Pharmacology, School of Medical Sciences, University of Cape CoastCape Coast, Ghana

**Keywords:** herbal medicines, adverse reactions, monitoring safety, challenges, public health

## Abstract

The use of herbal medicinal products and supplements has increased tremendously over the past three decades with not less than 80% of people worldwide relying on them for some part of primary healthcare. Although therapies involving these agents have shown promising potential with the efficacy of a good number of herbal products clearly established, many of them remain untested and their use are either poorly monitored or not even monitored at all. The consequence of this is an inadequate knowledge of their mode of action, potential adverse reactions, contraindications, and interactions with existing orthodox pharmaceuticals and functional foods to promote both safe and rational use of these agents. Since safety continues to be a major issue with the use of herbal remedies, it becomes imperative, therefore, that relevant regulatory authorities put in place appropriate measures to protect public health by ensuring that all herbal medicines are safe and of suitable quality. This review discusses toxicity-related issues and major safety concerns arising from the use of herbal medicinal products and also highlights some important challenges associated with effective monitoring of their safety.

## INTRODUCTION

The use of herbal medicines and phytonutrients or nutraceuticals continues to expand rapidly across the world with many people now resorting to these products for treatment of various health challenges in different national healthcare settings (WHO, 2004). This past decade has obviously witnessed a tremendous surge in acceptance and public interest in natural therapies both in developing and developed countries, with these herbal remedies being available not only in drug stores, but now also in food stores and supermarkets. It is estimated that up to four billion people (representing 80% of the world’s population) living in the developing world rely on herbal medicinal products as a primary source of healthcare and traditional medical practice which involves the use of herbs is viewed as an integral part of the culture in those communities (Mukherjee, 2002; Bodeker et al., 2005; Bandaranayake, 2006).

The use of herbal remedies has also been widely embraced in many developed countries with complementary and alternative medicines (CAMs) now becoming mainstream in the UK and the rest of Europe, as well as in North America and Australia (Committee on the Use of Complementary, and Alternative Medicine by the American Public, Board on Health Promotion, and Disease Prevention, Institute of Medicine, 2005; Calapai, 2008; Braun et al., 2010; Anquez-Traxler, 2011). In fact, while places like the UK have a historical tradition of using herbal medicines (Nissen, 2010), the use is also widespread and well established in some other European countries (Calapai, 2008). In these developed countries, the most important among many other reasons for seeking herbal therapy is the belief that it will promote healthier living. Herbal medicines are, therefore, often viewed as a balanced and moderate approach to healing and individuals who use them as home remedies and over-the-counter drugs spend huge amount of money (in excess of billions of dollars) on herbal products. This explains in part the reason sales of herbal medicines are booming and represents a substantial proportion of the global drug market (Roberts and Tyler, 1997; Blumenthal et al., 1998; WHO, 2002a; Kong et al., 2003; Pal and Shukla, 2003; WHO, 2005a; Bandaranayake, 2006).

As the global use of herbal medicinal products continues to grow and many more new products are introduced into the market, public health issues, and concerns surrounding their safety are also increasingly recognized. Although some herbal medicines have promising potential and are widely used, many of them remain untested and their use also not monitored. This makes knowledge of their potential adverse effects very limited and identification of the safest and most effective therapies as well as the promotion of their rational use more difficult (WHO, 2002b). It is also common knowledge that the safety of most herbal products is further compromised by lack of suitable quality controls, inadequate labeling, and the absence of appropriate patient information (Raynor et al., 2011). It has become essential, therefore, to furnish the general public including healthcare professionals with adequate information to facilitate better understanding of the risks associated with the use of these products and to ensure that all medicines are safe and of suitable quality. Discussion in this review is limited to toxicity-related issues and major safety concerns arising from the use of herbal medicines as well as factors promoting them. Some important challenges associated with effective monitoring of safety of these herbal remedies are also highlighted with a view to helping refocus relevant regulatory agencies on the need for effectiveness and ensuring adequate protection of public health and promoting safety.

## FACTORS RESPONSIBLE FOR INCREASED PATRONAGE AND SELF-MEDICATION WITH HERBAL MEDICINE

Essentially, herbal remedies consist of portions of plants or unpurified plant extracts containing several constituents which are often generally believed to work together synergistically. The recent resurgence of public interest in herbal remedies has been attributed to several factors some of which include (i) various claims on the efficacy or effectiveness of plant medicines, (ii) preference of consumers for natural therapies and a greater interest in alternative medicines, (iii) erroneous belief that herbal products are superior to manufactured products, (iv) dissatisfaction with the results from orthodox pharmaceuticals and the belief that herbal medicines might be effective in the treatment of certain diseases where conventional therapies and medicines have proven to be ineffective or inadequate, (v) high cost and side effects of most modern drugs, (vi) improvements in the quality, efficacy, and safety of herbal medicines with the development of science and technology, (vii) patients’ belief that their physicians have not properly identified the problem; hence the feeling that herbal remedies are another option, and (viii) a movement toward self-medication (Bandaranayake, 2006).

The increasing utilization of herbs for self-medication by patients or individuals is also attributed to a number of other reasons such as (i) patients being uncomfortable about discussing their medical problems and fear lack of confidentiality in handling their health information, (ii) fear of possible misdiagnosis and wrong treatment by patients with non-specific symptoms or general malaise, and (iii) lack of time to see a physician; this is usually a reason where prior visit did not yield any positive experience (Studdert et al., 1998). Furthermore, patients’ freedom of choice of a practitioner is also encouraging their utilization of alternative treatments and herbal remedies, although many select herbal medicines from a deductive approach based on anecdotal information, that is, “it worked for my friend or relative” (Parle and Bansal, 2006). So also, because of the influence of religion and greater level of spiritual consciousness, many individuals tend to be increasingly disposed to accepting therapeutic value of a treatment based on faith or intuition rather than scientific reasoning (Astin, 1998; Zeil, 1999). Herbal medicines, therefore, become particularly alluring when the body’s natural capacity for self-repair, given appropriate conditions, is emphasized (Parle and Bansal, 2006).

In addition to all these above-mentioned factors, the marketing strategies and efforts by various manufacturers of herbal medicines and their sales representatives have seriously projected these products into greater limelight. Various advertisements in the mass media including television and radio programmes have significantly increased consumers’ awareness and given the herbal products undue respectability and credibility (Brevort, 1998; Parle and Bansal, 2006). These advertisements are carefully presented to attract the different age groups of people that exist in the society. Children are encouraged to use herbs for their nutritional values to facilitate normal or healthy growth and development; young persons for their euphoric effects, supply essential ingredients to help them cope with daily stress and to prevent or slow the onset of aging; older persons for their anti-aging or rejuvenating effects and women for slimming and beauty enhancing effects (Parle and Bansal, 2006).

## INFLUENCE OF REGULATORY POLICIES ON SAFETY OF HERBAL MEDICINES

It has been observed that most of the problems associated with the use of traditional and herbal medicines arise mainly from the classification of many of these products as foods or dietary supplements in some countries. As such, evidence of quality, efficacy, and safety of these herbal medicines is not required before marketing. In the same vein, quality tests and production standards tend to be less rigorous or controlled and in some cases, traditional health practitioners may not be certified or licensed. The safety of traditional and herbal medicines has therefore become a major concern to both national health authorities and the general public (Kasilo and Trapsida, 2011).

Until 2011, there were three possible regulatory routes by which an herbal product could reach a consumer in the UK. The unlicensed herbal remedy is the commonest route which does not have to meet specific standards of safety and quality neither is it required to be accompanied by safety information for the consumer (Raynor et al., 2011). Recently, the European Union (EU) implemented a directive after a 7-year transition period to harmonize the regulation of traditional herbal medicine products across the EU and establish a simplified licensing system in order to help the public make informed choices about the use of herbal products. This requires that all manufactured herbal products either gain a product license of the type needed to manufacture “conventional” products or become registered as a “traditional herbal medicinal product” (Routledge, 2008; Raynor et al., 2011).

Like conventional medicines, licensed herbal medicines hold a product license based on safety, quality, and efficacy. Hence, it is compulsory that they are accompanied by comprehensive information such as indications, precautions, how to use the product, side effects, how to store the product and regulatory information, for safe use. This information is usually provided on a leaflet inserted into the product package (Raynor et al., 2011). On the other hand, due to insufficient evidence of reproducible efficacy to meet regulatory standards, license cannot be obtained for some herbal medicines to sell these products. This led to the creation of a new category of traditional herbal registration (THR) with a transition period of seven years (European Union Herbal Medicines Directive, 2004). In line with this, the Traditional Herbal Medicines Registration Scheme, which is a “simplified registration scheme,” was introduced in the UK. In this scheme, herbal medicinal products are required to meet specific standards of safety and quality, agree upon indications for use based on their traditional use and also provide information in a leaflet to promote safe use of the product by the purchaser (Raynor et al., 2011). However, this is not the case in many other parts of the world, especially in the developing countries where many unregistered and poorly regulated herbal products are sold freely on the market with little or no restraint. Furthermore, the common misconception that natural products are not toxic and are devoid of adverse effects often lead to improper use and unrestrained intake and this has also resulted in severe poisoning and acute health problems. This misconception is not limited to the developing countries. It also exists in highly developed countries, where the general public often resorts to “natural” products without any proper awareness or information on the associated risks, particularly in the event of excessive or chronic use (UNESCO, 2013).

## TOXICITY AND ADVERSE HEALTH EFFECTS OF SOME COMMON HERBAL MEDICINES

In most countries, herbal medicines and related products are introduced into the market without any mandatory safety or toxicological evaluation. Many of these countries also lack effective machinery to regulate manufacturing practices and quality standards. These herbal products are continuously made available to consumers without prescription in most cases and the potential hazards in an inferior product are hardly recognized (Bandaranayake, 2006).

It is important to reiterate the staggering rate at which interest and use of herbal medicines is expanding. Over the past decade, the use of herbal medicines represents approximately 40% of all healthcare services delivered in China while the percentage of the population which has used herbal medicines at least once in Australia, Canada, USA, Belgium, and France is estimated at 48%, 70%, 42%, 38%, and 75%, respectively (Foster et al., 2000; WHO, 2002b). In spite of the positive perception of patients on the use of herbal medicines and alleged satisfaction with therapeutic outcomes coupled with their disappointment with conventional allopathic or orthodox medicines in terms of effectiveness and/or safety (Huxtable, 1990; Abbot and Ernst, 1997), the problem of safety of herbal remedies continues to remain a major issue of concern.

The general perception that herbal remedies or drugs are very safe and devoid of adverse effects is not only untrue, but also misleading. Herbs have been shown to be capable of producing a wide range of undesirable or adverse reactions some of which are capable of causing serious injuries, life-threatening conditions, and even death. Numerous and irrefutable cases of poisoning have been reported in the literature (Vanherweghem and Degaute, 1998; Cosyns et al., 1999; Ernst, 2002). The toxicity evaluation of the polyherbal formula, Yoyo “Cleanser” Bitters®, conducted recently in our laboratory (Ekor et al., 2010), was prompted by an unpublished case report of a young male adult who had been on self-medication with this herbal product and was subsequently admitted to the hospital on account of liver failure. Yoyo “Cleanser” Bitters® is one of the herbal remedies that is widely advertised in the various Nigerian media and as such has gained so much public acceptance over time and continues to enjoy increased patronage among consumers, especially in the southwestern part of the country. Our study revealed that this herbal formula was capable of elevating plasma levels of liver enzymes and inducing hypokalemia following 30 days administration in rats. From our observation, the potassium loss (which is capable of predisposing to dangerous arrhythmias) was a greater risk associated with this herb during this sub-acute exposure or toxicity study. Prior to this study, we had evaluated the safety of “super B blood purifier” and “super B seven keys to power” mixtures in experimental model over a decade ago (John et al., 1997). These herbal mixtures were marketed by a registered Nigerian company which cultivated medicinal plants and manufactured medicinal herbal preparations. The herbal blood tonics were well patronized by common folks who claim their efficacy according to the manufacturer’s stipulation that “they are safe, give strength and cleanse the blood and body of infection.” We obtained the herbal constituents (*Entandrophragma utile* and *Anacardium occidentalis*) and investigated the individual plant extract as well as the herbal tonics made from them. Although, all the extracts and tonics proved safe during acute toxicity study, chronic toxicity testing revealed splenic enlargement in 10% of mice that received *E. utile* or either of the two tonics and one case of lung tumor (John et al., 1997). Recently, Auerbach et al. (2012) reported an association between traditional herbal medicine use and the development of liver fibrosis among study participants in Uganda. A number of Chinese herbal medicines and other herbal medicines from different parts of the world have also been implicated in cases of poisoning. Many of them have been shown to contain toxic compounds which are capable of reacting with cellular macromolecules including DNA, causing cellular toxicity, and/or genotoxicity (Rietjens et al., 2005). For the purpose of brevity and other obvious constraints, adverse reactions of only a few commonly used herbal medicines are described below.

### ARISTOLOCHIC ACIDS AND *Aristolochia* SPECIES

Following the discovery of the nephrotoxic and carcinogenic potentials of aristolochic acids (Vanherweghem et al., 1993), several studies confirmed their genotoxic activity (Kohara et al., 2002; Fang et al., 2011; Hwang et al., 2012). Schmeiser et al. (1996) demonstrated the presence of aristolochic acids-related DNA adducts in renal tissues of patients. These mutagenic adducts when formed are usually poorly repaired (Sidorenko et al., 2012) and are capable of persisting for years in DNA (Nortier et al., 2000). Aristolochic acids I and II have been identified in different Asian medicinal plants and also reported to be present in slimming products. This has led to the banning of medicinal products containing these acids in Belgium, UK, Canada, Australia, and Germany (Hashimoto et al., 1999; Lee et al., 2002; Zhou et al., 2013).

Misidentification of medicinal plants and mislabeling herbal medicinal products are sometimes responsible for some of the observed adverse events or interaction and that is the reason it is important to assess herbal medicines for possible presence of adulterants. In the UK and many other countries *Aristolochia fangchi* was linked to the development of subacute interstitial fibrosis of the kidney referred to as “Chinese herbs nephropathy” (Cosyns et al., 1994; Tanaka et al., 1997a,b; Stengel and Jones, 1998; Lord et al., 1999). Also, recently a 75-year-old man was reported to have died in Australia from kidney failure associated with a toxic preparation containing the root of *Aristolochia fangchi* which he purchased over the internet for psoriasis (Chau et al., 2011). This case report suggested the chronic use of aristolochic acid-containing herbal product as the most likely cause of the patient’s death. Similar cases had previously been reported in Taiwan and Japan (Yang et al., 2002; Hong et al., 2006). Consumption of aristolochic acid-containing Chinese herbal products has also been demonstrated in several studies to be associated with increased risk of urothelial cancer (Li et al., 2008; Yang et al., 2009; Lai et al., 2010; Chen et al., 2012).

A few years back, poisoning that was attributed to Fang-Ji (*Stephania tetrandra* S. Moore) in a weight-loss preparation (Cosyns et al., 1994; van Ypersele de Strihou, 1995; Reginster et al., 1997; Kessler, 2000) was actually caused by Guang-Fang-Ji (*Aristolochia fangchi*; Vanhaelen et al., 1994). The presence of aristolochic acids in the latter produced dramatic adverse reactions which led to nephrotoxic and carcinogenic events in more than 100 women using this weight-loss preparation (Zhou et al., 2004). In this instance, the similarity in the names of the two herbal products was responsible for the confusion and the unfortunate events.

### Ephedra sinica

*Ephedra* is a very popular herb with long history of traditional use in respiratory conditions (Zhang et al., 1999; Bensky et al., 2004). This herb, whose efficacy has been demonstrated in a number of randomized double-blind clinical trials (Boozer et al., 2002; Haller et al., 2005; Kim et al., 2008), is currently included in the Chinese Pharmacopoeia for therapeutic use and classified as non-toxic. It is an ingredient in commonly used formulary preparations such as Xiaoqinglong Heji for common cold and Zhisou Dingchuan Koufuye for asthma (Chinese Pharmacopoeia Commission, 2010). *Ephedra* has been marketed in the US as a weight-loss dietary supplement and its use associated with a number of serious cardiovascular and central nervous systems (CNSs) adverse effects (Verduin and Labbate, 2002; Hackman et al., 2006; Hallas et al., 2008; Chen et al., 2010). Several case reports have also linked the use of *Ephedra sinica* and *Ephedra*-containing dietary supplements to adverse events such as hepatotoxicity (Skoulidis et al., 2005; Schoepfer et al., 2007), neurotoxicity (Varlibas et al., 2009), and transient blindness (Moawad et al., 2006).

### *Aconitum* SPECIES

*Aconitum carmichaeli* and *Aconitum kusnezoffii* are used traditionally for pain relief (Chinese Pharmacopoeia Commission, 2010). The toxicity of the medicinal plants derives primarily from the presence of diester diterpene alkaloids such as aconitine, mesaconitine, and hypaconitine in them (Xu et al., 2005). These medicinal plants constitute important ingredients in some commonly used herbal preparations like Sini Tang, Fuzi Lizhong Wan, and Guifu Dihuang Wan for stroke and heart failure, diarrhea and diabetes, respectively (Chinese Pharmacopoeia Commission, 2010). Poisoning due to homemade medicated liquor containing aconite and traditional medicine containing *A. carmichaeli* were reported in China between 1999 and 2008 (Liu et al., 2011). Severe cases of cardiac toxicity from consumption of aconitine-containing herbal preparation manifesting as ventricular tachycardia and fibrillation and eventually leading to death have been reported (Tai et al., 1992; Fujita et al., 2007; Dhesi et al., 2010; Lin et al., 2011). In other studies bradycardia and hypotension were observed (Chan, 2009). Clinical cases of aconitine poisoning from *A. kusnezoffii* consumption have also been reported (Chan and Critchley, 1994; Chan, 2002). Furthermore, the toxic effect of the combination of *A. kusnezoffii* and *A. carmichaeli* overdose which manifested as bradycardia and hypotension was also reported by Chan (2009) in a case study.

### Tussilago farfara

Traditionally, *Tussilago farfara* has been used effectively for thousands of years for the treatment of acute and chronic cough and it is generally regarded as non-toxic (Chinese Pharmacopoeia Commission, 2010). Total alkaloids and senkirkine isolated from this plant, on the other hand, have been demonstrated to be hepatotoxic (Zhang et al., 2008). Recently, the potential health effects of the pyrrolizidine alkaloids found in *T. farfara* was reviewed and hepatic veno-occlusive disease and cirrhosis were suggested as the major potential disease outcome in human (Edgar et al., 2011). However, restrictions in intake of pyrrolizidine-containing herbs and further investigations were recommended because of paucity of data on toxicity assessment in human (Kim et al., 2013).

### GARLIC (*Allium sativum*)

Garlic has found relevance for management of hypertension and hypercholesterolemia besides its use as a food or food additive. It is known to contain alliin, which on crushing or chopping in the absence of heat or acid becomes activated by alliinase to allicin. Adverse effects associated with garlic extract including burning sensation in the gastrointestinal tract, nausea, diaphoresis, and lightheadedness have been reported. This extract may also cause contact dermatitis and morbid spontaneous spinal epidural hematoma has been attributed solely to excess garlic ingestion (Rose et al., 1990).

### *Ginkgo biloba* AND *Ginseng*

*Ginkgo biloba* has found widespread use in a variety of conditions and several products such as elixirs, extracts, tea, as well as capsules and tablets that may differ in terms of content, have been made from the dried root (Sparreboom et al., 2004). The whole root which contains ginsenosides is usually used because these compounds possess specific pharmacologic effects that may oppose each other (Chong and Oberholzer, 1988). Over 30 ginsenosides have been identified and these compounds are being investigated for their ability to inhibit cell proliferation, tumor cell invasion, and/or metastasis (He et al., 2011; Kim et al., 2012). Recently, the ability of ginsenosides to modulate signaling pathways involving cell cycle, inflammatory, or growth factor pathways, was demonstrated (Nag et al., 2012; Dunnick and Nyska, 2013). The leaf extracts of ginkgo had also been demonstrated to contain active compounds that had found usefulness in improving circulation and cognition (Boullata and Nace, 2000).

The plant extracts appear to be relatively safe, although headache, dizziness, restlessness, nausea, vomiting, diarrhea, and dermal sensitivity are the most common side effects that have been observed. *Ginkgo* has been demonstrated to be capable of inhibiting platelet-activating factor and altering bleeding times. Therefore, cautious use had been advised in individuals or patients on anticoagulants therapy (Boullata and Nace, 2000). The ability of ginkgo to induce liver cancer in experimental model was reported recently and genotoxic mechanisms were suggested to play some role in the carcinogenic process (National Toxicology Program, 2012; Dunnick and Nyska, 2013). Similar observation was made in the thyroid gland and further studies are required to determine whether the mechanisms for the ginkgo-induced thyroid tumors are also found in humans (National Toxicology Program, 2012; Dunnick and Nyska, 2013). In addition to the carcinogenic effects in the liver and thyroid, ginkgo has also been shown to be capable of inducing tumorigenesis in the nasal cavity. The pathogenesis of the nasal lesions was suggested to be related to toxic metabolites of ginkgo reaching the nasal cavity from the systemic circulation (Reed, 1993; Sells et al., 2007). Some other authors, however, have attributed the nasal lesions to gastric reflux/post gavage reflux through the nasopharyngeal duct (Damsch et al., 2011a,b).

Although, *Ginseng* products as well as the published descriptions rarely report or specify the species, *Ginseng* used as herbal remedies has different botanical sources generally obtained from several *Panax* species including *Panax quinquefolius, Panax Ginseng*, and *Panax pseudoginseng*. In large or excessive doses, *Ginseng* has been reported to cause agitation, insomnia, and elevation of blood pressure while mastalgia and vaginal bleeding have been observed at recommended doses (Baldwin et al., 1986; Dunnick and Nyska, 2013). Other adverse effects that have reported include transient nervousness, excitation, insomnia, inability to concentrate, headache, epistaxis, and allergies. A case of severe but non-fatal Stevens–Johnson syndrome was reported following a 3-day *Ginseng* administration (Dega et al., 1996). Also, spontaneous bleeding from the iris into the anterior chamber of the eye was linked to the use of *G. biloba* extract (Rosenblatt and Mindel, 1997).Separate case reports had also linked the use of *G. biloba* to the development of bilateral subdural hematomas and subarachnoid hemorrhage (Rowin and Lewis, 1996; Vale, 1998).

### KAVA (*Piper methysticum*)

Kava is known for its CNS depressant activity and it is commonly used as an anxiolytic agent. Commonly reported side effects of this medicinal plant include headache, dizziness, gastrointestinal discomfort, and localized numbness after oral ingestion. Large dosages has been shown to be capable of giving rise to dry, scaly skin, and yellowish discoloration of the skin and nails, photosensitivity and redness of the eye. Excessive consumption of kava may also lead to photophobia and diplopia (Boullata and Nace, 2000). The muscle relaxant and anticonvulsant effects of this plant have been attributed to its active pyrones constituent. Significant interaction of kava with other CNS depressants which may lead to a comatose state has been reported and as such concurrent use with these agents is usually not advised (Almeida and Grimsley, 1996; Wong et al., 1998). In 2002, the US Food and Drug Administration received several reports of liver-related injuries alongside that of a previously healthy young female who eventually required liver transplantation following consumption of kava-containing products. This prompted the FDA to alert consumers on the potential risk of severe liver injury associated with the use of kava-containing dietary supplements. This liver-related risks also prompted regulatory agencies in other countries including Germany, Switzerland, France, Canada, and the UK to warn consumers about the potential risks of kava use and also went on to consider the withdrawal of kava-containing products from the market (U.S. Food and Drug Administration, 2002). In over 25 reports of adverse events outside the US, kava-containing products were associated with liver-related injuries like hepatitis, cirrhosis, and liver failure with some of the affected patients eventually requiring liver transplants (U.S. Food and Drug Administration, 2002).

### ST. JOHN’S WORT

*Hypericum perforatum,* popularly known as St. John’s wort, contains active compounds, such as hypericin, hyperforin, and melatonin. It was once used against viral infections but has gained increased use for mild depressive symptoms (Bennett et al., 1998; Rey and Walter, 1998). Adverse effects reportedly associated with its use include allergic reactions, headache, dizziness, restlessness, fatigue, dry mouth, nausea, vomiting, constipation, and photosensitivity. Hyperesthesia and a syndrome of dyspnea and hyperventilation with flushing headache, mydriasis, nausea, palpitations, and tremor, have been reported (Rey and Walter, 1998). Schneck (1998) reported a hypomanic episode in a patient with a history of panic disorder who took St. John’s wort tincture for 10 days. The hypomanic episode, however, resolved within 2 days of discontinuing the herbal remedy. Interaction of St. John’s wort with antidepressants and anticoagulants has been demonstrated and the herbal remedy is usually not recommended in pregnancy because of its uterotonic activity (Rey and Walter, 1998).

## CHALLENGES ASSOCIATED WITH MONITORING SAFETY OF HERBAL MEDICINES

With the enormous global consumption of herbal products and medicines, it is high time they were included in pharmacovigilance systems. In terms of population exposure alone, it is essential to identify the risks associated with the use of herbal medicines, and in this regard, the safety of these products has become an issue of great public health importance (WHO, 2004, 2005b). There is no doubt that the increasing cases of poisoning associated with the use herbal medicines in many parts of the world in recent times, is necessitating the need to ensure thorough toxicity assessment alongside active pharmacovigilance on these products in order to promote their safe use and protect public health (Zhou et al., 2013).

The development as well as implementation of the regulation of traditional or herbal medicines in different parts of the world is often confronted with several challenges. Challenges often encountered and common to many countries are those related to regulatory status, assessment of safety and efficacy, quality control, safety monitoring and inadequate or poor knowledge about traditional, complementary/alternative, and herbal medicines within national drug regulatory authorities (WHO, 2005b).

### CHALLENGES RELATED TO THE REGULATORY STATUS OF HERBAL MEDICINES

The definition and categorization of herbal medicines vary from one country to another. Depending on the regulations applying to foods and medicines, a single medicinal plant may be categorized as a food, a functional food, a dietary supplement, or a herbal medicine in different countries. This introduces serious difficulty in the definition of the concept of herbal medicines for the purposes of national drug regulation while at the same time also confusing patients and consumers (WHO, 2005b). In the United States, for example, natural products are regulated under the Dietary Supplement Health and Education Act (DSHEA) of 1994 (U.S. Food and Drug Administration, 2012). By definition, a dietary supplement is a product that is ingested and is intended to supplement the diet and contains a “dietary ingredient.” The dietary ingredients in these products may include vitamins, minerals, herbs, or other botanicals (U.S. Food and Drug Administration, 2011). Under the DSHEA, additional toxicity studies are generally not required if the herb has been on the market prior to 1994 (National Institute of Health (NIH) Office of Dietary Supplements, 2011). In this regard, the FDA bears the burden to prove that a herbal medicinal product or “dietary ingredient” is toxic or not safe for use. Additional major challenge in many countries is the fact that regulatory information on herbal medicines is often not shared between regulatory authorities and safety monitoring or pharmacovigilance centers (WHO, 2004).

### CHALLENGES RELATED TO THE ASSESSMENT OF SAFETY AND EFFICACY

There is no gainsaying the fact that the requirements as well as the research protocols, standards and methods needed for the evaluation of the safety and efficacy of herbal medicines are much more complex than those required for conventional or orthodox pharmaceuticals (WHO, 2005b; Zhou et al., 2013). A single herbal medicine or medicinal plant may contain hundreds of natural constituents, and a mixed herbal medicinal product may contain several times that number. Suppose every active ingredient were to be isolated from individual herb from which the herbal medicine is formulated or produced, the time and resources required would be tremendous. Such an analysis may practically be impossible especially where an herbal product is a mixture of two or more herbs (WHO, 2005b).

### CHALLENGES RELATED TO QUALITY CONTROL OF HERBAL MEDICINES

The quality of the source materials used in the production of herbal medicines determines to a large extent the safety and efficacy of these herbal remedies. Generally, the quality of source materials is dependent not only on intrinsic (genetic) factors, but also on extrinsic factors like environmental conditions, good agricultural, and good collection practices (GACP) for medicinal plants, including plant selection and cultivation. It is the combination of these factors that makes it difficult to perform quality controls on the raw materials of herbal medicines (WHO, 2004, 2005b). According to good manufacturing practice (GMP), correct identification of species of medicinal plants, special storage, and special sanitation and cleaning methods for various materials are important requirements for quality control of starting materials.

One of the major challenges often encountered in the quality control of finished herbal medicinal products, especially mixture herbal products, is the difficulty in ascertaining the inclusion of all the plants or starting materials (WHO, 2005b). Thus, the general requirements and methods for quality control of finished herbal products remain far more complex than for other pharmaceuticals (WHO, 2003, 2004, 2005b). To ensure safety and efficacy of herbal medicines, therefore, WHO continues to recommend the institution of quality assurance and control measures such as national quality specification and standards for herbal materials, GMP for herbal medicines, labeling, and licensing schemes for manufacturing, import and marketing, in countries where herbal medicines are regulated (WHO, 2004).

### CHALLENGES RELATED TO SAFETY MONITORING OF HERBAL MEDICINES

In recent years, issues relating to increasing use of herbal products in developed countries, dependence of many people living in developing countries on plants as a major source of medicines coupled with absence or weak regulation of herbal medicines in most countries and the occurrence of high-profile safety concerns, have increased awareness of the need to monitor safety and deepen understanding of possible harmful as well as potential benefits associated with the use of herbal medicines (Rodrigues and Barnes, 2013). Adverse events arising from consumption of herbal medicines are attributable to several factors among which include the use of the wrong species of plant by mistake, adulteration of herbal products with other, undeclared medicines, contamination with toxic or hazardous substances, overdosage, misuse of herbal medicines by either healthcare providers or consumers and use of herbal medicines concomitantly with other medicines.

Although, the assessment of the safety of herbal medicines has become an important issue for consumers, regulatory authorities, and healthcare professionals, analysis of adverse events related to the use of these products is much more complex than in the case of conventional pharmaceuticals (WHO, 2005b; Zhou et al., 2013). It is also recognized that evaluation of safety is complicated by factors such as the geographical origin of plant material, different processing techniques, route of administration, and compatibility with other medicines (Zhang et al., 2012). Furthermore, there is lack of the knowledge and/or poor emphasis on the importance of taxonomic botany and documentation by most manufacturers of herbal medicines and this poses peculiar challenges during identification and collection of medicinal plants used for herbal remedies (Farah et al., 2000). In order to eliminate the confusion created by the common names, it is necessary to adopt the most commonly used binomial names (including their binomial synonyms) for medicinal plants. For example, *Artemisia absinthium* L., which contains an active narcotic derivative and capable causing CNS disorders and generalized mental deterioration, has at least 11 different common names. Seven of the common names bear no resemblance to its botanical name. Because common names are mainly used, *Heliotropium europaeum* (heliotrope), which contains potent hepatotoxic pyrrolidine alkaloids, is often confused with *Valerian officinalis* (garden heliotrope), known to contain valepotriates with sedative and muscle relaxant properties. This explains why it is important to provide the exact scientific name of the plant, the plant part used, and the name of the manufacturer when reporting adverse drug reactions of herbal medicines. Therefore, effective monitoring of safety of herbal medicine will require effective collaboration between botanists, phytochemists, pharmacologists, and other major stake-holders.

## CONCLUSION AND RECOMMENDATIONS

The global acceptance and use of herbal medicines and related products continue to assume exponential increase. Issues relating to adverse reactions in recent times are also becoming more vivid, increasing in prevalence and no longer debatable because of previous misconception of regarding or categorizing herbal medicinal products as “safe” because they are derived from “natural” source. The reality is that “safety” and “natural” are not synonymous. Therefore, regulatory policies on herbal medicines need to be standardized and strengthened on a global scale. Relevant regulatory authorities in different countries of the world need to be proactive and continue to put in place appropriate measures to protect public health by ensuring that all herbal medicines approved for sale are safe and of suitable quality.

Providers of medicines, such as physicians, nurses, and pharmacists, often have little training in and understanding of how herbal medicines affect the health of their patients. Many of them are also poorly informed about these products and how they are being used. Adequate training is now very essential since most patients are almost often on other types of prescription or non-prescription medicines. In spite of the fact that the active involvement of orthodox healthcare professionals is continuously solicited and huge responsibility lies with them in terms of their valuable contributions to safety monitoring of medicinal products, it is also very important that all providers of herbal medicines are sufficiently empowered to play a role in monitoring safety of herbal medicines. This, however, should be in collaboration with the orthodox healthcare professionals. For this to be effective, it would be essential to create an atmosphere of trust to facilitate adequate sharing of knowledge about the use and safety of herbal medicines. In fact, the education of healthcare professionals, providers of herbal medicines, and patients/consumers is vital for the prevention of potentially serious risks from misuse of herbal medicines.

Of crucial importance also is an appropriate knowledge base relevant to diagnostic and treatment decision-making. Furthermore, individual healthcare provider should also show sufficient commitment toward understanding the use of herbal medicines. This can be by asking relevant questions about the use of these herbal remedies among others whenever they encounter patients who are taking these medications. Health professionals who work in poisons centers and health information services also need to be informed about herbal medicines. Finally, as with other medicines for human use, it has become mandatory that herbal medicines are covered in every country of the world by a drug regulatory framework to ensure that they conform with required standards of safety, quality, and efficacy.

## Conflict of Interest Statement

The author declares that the research was conducted in the absence of any commercial or financial relationships that could be construed as a potential conflict of interest.
